# Case Report: Two cases of pathologic complete response after upfront chemoimmunotherapy for initially unresectable gastric cancer

**DOI:** 10.3389/fonc.2026.1877888

**Published:** 2026-08-26

**Authors:** Megan Smith-Uffen, Prathana Nathan, Chris J. Zhang, Ken Leslie, Leah McDonald, Matthew Cecchini, Mark Vincent

**Affiliations:** 1 Medical Oncology Postgraduate Training Program, Western University, London, ON, Canada; 2 Department of Surgery, London Health Sciences Center, London, ON, Canada; 3 Department of Pathology and Laboratory Medicine, London Health Sciences Centre, London, ON, Canada; 4 Division of Medical Oncology, London Health Sciences Center, London, ON, Canada

**Keywords:** case report, chemoimmunotherapy, complete pathological response, gastric cancer, resectability

## Abstract

Gastric cancer causes considerable morbidity and mortality. Chemoimmunotherapy has improved survival in the metastatic setting and pathologic complete response (pCR) for those with resectable disease. However, the ability of chemoimmunotherapy to convert locally advanced unresectable into resectable disease, or to lead to a pCR in this setting, is not known. Here we describe two cases of patients who were initially diagnosed with unresectable and incurable gastric cancer who, after receiving upfront chemoimmunotherapy, were converted to resectable disease, received curative surgery, and demonstrated a pCR. Both patients had microsatellite stable, HER2-negative gastric cancers with a combined positive PD-L1 score of greater than or equal to 10. Both experienced an immunotherapy-related adverse event and are currently on surveillance. These cases highlight the importance of reassessing candidacy for surgical resection among those with a good clinical response to upfront treatment, and the growing role of chemoimmunotherapy in the treatment of incurable and curable gastric cancer.

## 1 Introduction

Gastric, esophageal, and gastroesophageal junction (GEJ) cancers are a heterogenous group of cancers that cause considerable morbidity and mortality worldwide. Gastric cancer is the fifth most common malignancy and the third most common cause of cancer-related death (1). It is responsible for approximately 8.2% of all cancer-related deaths worldwide; the highest mortality and incidence rates are found in East Asia (1). The current standard of care for locally advanced, upfront resectable gastric cancer is perioperative combination chemotherapy with 5-fluorouracil, leucovorin, oxaliplatin, and docetaxel (FLOT) (2). For patients who undergo upfront resection but are deemed locally advanced at the time of surgery, adjuvant chemotherapy or chemoradiation can be considered (3–5). Regrettably, less than 60% of patients are alive 3 years later with either approach (6).

In the metastatic setting, gastric cancer is treated similarly to esophageal and GEJ primaries – with treatment choice driven by biomarker status. Patients with HER2-expressing tumors receive a combination of HER2-targeted therapy and chemotherapy (7); those with deficient mismatch repair (dMMR) or positive programmed death-ligand 1 (PD-L1) scores receive a combination of immunotherapy and chemotherapy (8–11); and those with both HER2 expression and PD-L1 positivity receive a combination of immunotherapy, HER2-targeted therapy, and chemotherapy (12). Patients with CLAUDIN 18.2 (CLDN18.2) positive tumors may now receive a CLDN18.2-targeted monoclonal antibody in combination with chemotherapy; and therapies targeting fibroblast growth factor receptor 2 (FGFR2) are under development (13). Biomarker-negative patients receive chemotherapy alone, typically with 5-fluorouracil, leucovorin, and oxaliplatin (FOLFOX).

Immunotherapy has revolutionized the field of oncology, with improved survival and often tolerable side effect profiles across many tumor sites. In gastric cancer, immune checkpoint inhibitors (ICIs) are standard of care in the metastatic setting for patients with a PD-L1 combined positive score (CPS) of greater than 1. This is informed by multiple trials that have shown a survival benefit for these patients (8–11). For resectable disease, nivolumab is used in the adjuvant setting for locally advanced esophageal or GEJ cancers with residual pathologic disease after esophagectomy (14). However, gastric cancer patients were excluded from this trial.

ICIs are also under investigation in the neoadjuvant and perioperative setting. An interim analysis of the phase II/III DANTE/IKF-s633 trial demonstrated improved pathologic complete regression rates for resectable esophagogastric carcinoma with chemotherapy and atezolizumab (24% vs 15% with chemotherapy alone, P = 0.032), a difference that was more pronounced in the CPS ≥10 subgroup (33% vs 12%) (15). A smaller trial demonstrated improved gastric R0 resection rate and pathologic complete response (pCR) with camrelizumab in a similar group of patients (16). The phase III Keynote-585 trial found a significant improvement in pCR for patients with resectable locally advanced gastric and GEJ treated with pembrolizumab and chemotherapy, but this did not translate into a significant survival benefit (17). However, most patients in this trial received a cisplatin-based doublet chemotherapy backbone, which is not the standard of care. In an interim analysis of a subgroup who received FLOT, event free survival (EFS) and overall survival (OS) were not reached (18).

Finally, MATTERHORN, a phase III trial comparing FLOT with/without durvalumab in resectable gastric and GEJ cancer, showed an improvement in pCR rates and downstaging (19), a significant improvement in EFS and what appeared to be a significantly improved OS among patients at 12 months and onward, although there was no significant difference in OS in the 0 to 12 month interval (20).

It should be noted that dMMR gastric cancers are a unique subset with a high tumor mutational burden that is treated preferentially with immunotherapy in the metastatic setting. Trials are ongoing to evaluate the role of perioperative immunotherapy in these patients, which is outside the scope of this paper (21). Gastric cancers caused by Epstein-Barr Virus (EBV) are associated with PD-L1 overexpression and may be similarly responsive to immunotherapy, although EBV status is not currently performed as a standard biomarker (22).

Chemoimmunotherapy has improved survival in the metastatic setting and pathologic response among those with resectable disease. However, the ability of palliative-intent chemoimmunotherapy to convert unresectable, locally advanced disease to resectable disease, or to drive a pathologic complete response in this setting, is not well known. Here we describe two cases of patients who were initially diagnosed with nonmetastatic but locally unresectable gastric cancer who, after receiving upfront chemoimmunotherapy, were converted to resectability, received curative-intent surgery, and demonstrated a pCR.

## 2 Detailed case description

### 2.1 Case 1:

**Figure 1:**
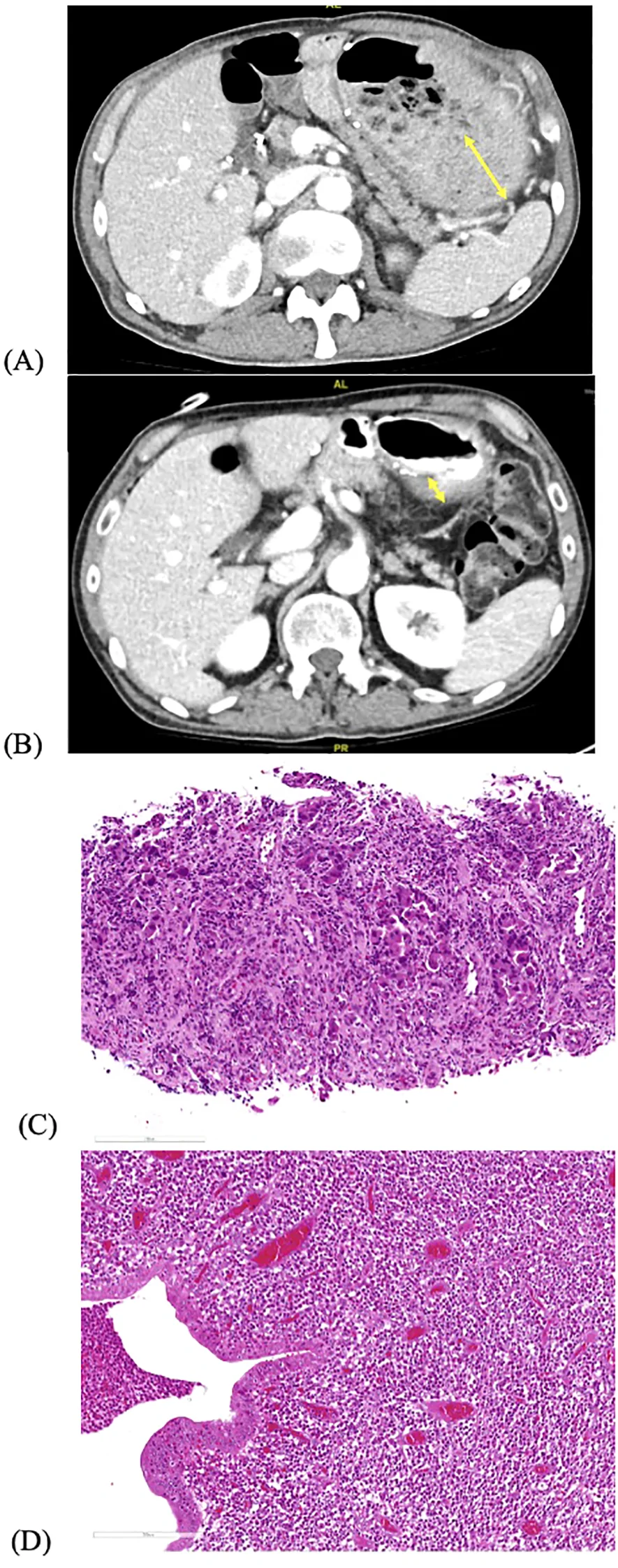
CT images in the **(A)** preoperative setting and after chemoimmunotherapy **(B)**. Note that the patient has had a prior surgical resection and Billroth type 2 anastomosis for peptic ulcer disease. **(C)** Hematoxylin and eosin (H&E)-stained section of the pretreatment endoscopic biopsy from the gastric mass showing poorly differentiated adenocarcinoma composed of pleomorphic malignant epithelial cells arranged in irregular clusters and as discohesive single cells within a prominent lymphoplasmacytic inflammatory background. **(D)** H&E-stained representative section of the treated tumor bed from the gastrectomy specimen showing reactive stromal change and dense chronic inflammation, with no viable carcinoma identified.

A 70-year-old male with a history of chronic peptic ulcer disease presented with weight loss and intermittent melena (**Table 1**). A CT of the abdomen and pelvis showed an ulcerating stomach mass measuring 7.3 cm by 4.8 cm, contacting and potentially invading the splenic flexure (**Figure 1A**). His biopsy revealed poorly differentiated gastric adenocarcinoma, pMMR, HER2-negative, CPS ≥10, and EBV-positive (**Figure 1C**). He underwent diagnostic laparoscopy, during which possible involvement of the splenic flexure of the colon was identified. There was no gross peritoneal disease, and pathology was negative for malignancy. On the basis of his diagnostic imaging and intraoperative findings, he was diagnosed with T4N0M0 disease with invasion into the mid-to-distal transverse colon. The T stage was not confirmed with biopsy or endoscopic ultrasound.

**Table 1:** Timeline of events in case 1.

Date	Event
May 2024	Admitted with anemia, weight loss and melena stools, diagnosis confirmed via endoscopic biopsy
June 2024	Diagnostic laparoscopy with washings
July 2024	Started FOLFOX plus nivolumab
September 2024	Immune checkpoint inhibitor related hepatitis
October 2024	CT showing marked reduction in gastric mass and tumor board discussion
November 2024	Underwent gastrectomy with pCR

He was deemed ineligible for upfront curative-intent surgery due to the locally advanced nature of his disease which would have required multivisceral resection, compounded by his poor performance status and significant weight loss. Upfront resection would have required a total gastrectomy with en bloc transverse colectomy (extended right colectomy). The treating team felt that systemic therapy offered the patient the best opportunity for prolonged survival and controlling symptoms. He received nivolumab in combination with FOLFOX chemotherapy as first-line treatment.

Treatment was interrupted by grade 3 immunotherapy-induced hepatitis. Nivolumab was discontinued following this and the hepatitis subsequently resolved within one month with no recurrence of hepatitis. Overall, he only received 3 doses of nivolumab and 8 doses of FOLFOX. A follow-up CT of the chest, abdomen and pelvis revealed marked reduction in the size of the gastric mass with only a small amount of residual thickening remaining (**Figure 1B**). He experienced significant improvement in his performance status. His case was discussed at multidisciplinary tumor boards, and it was decided that he was now a candidate for surgery given the CT results and his improved performance status. He underwent a total gastrectomy with esophagojejunostomy and Roux-en-Y procedure. The tumor bed was extensively sampled with 8 blocks of potential tumor and 26 lymph nodes with cytokeratin AE1/AE3 performed showing no viable residual tumor. (**Figure 1D**). He remains on surveillance.

### 2.2 Case 2:

**Figure 2:**
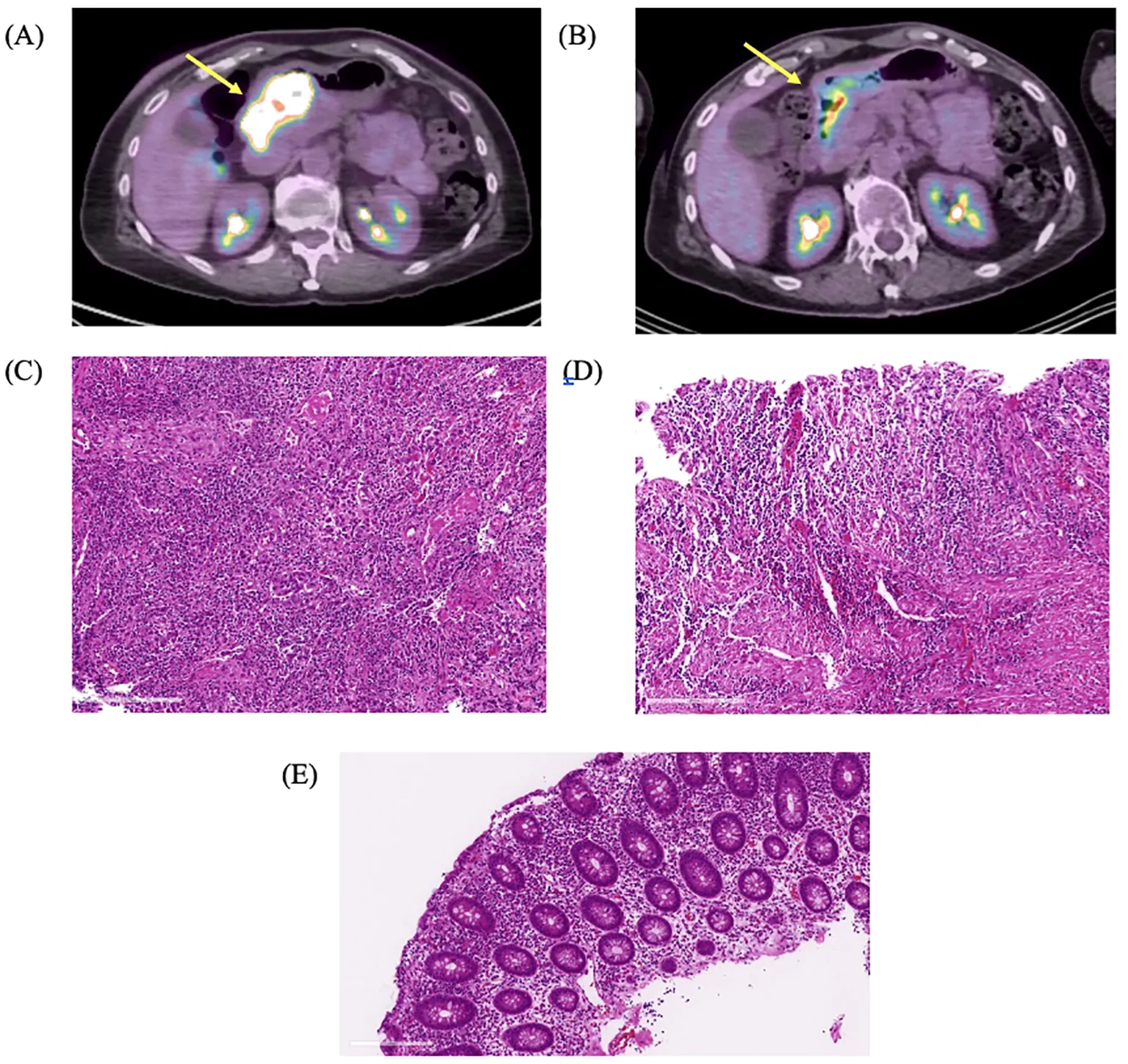
**(A)** PET images of the avid gastric mass; **(B)** PET images showing minimal avidity after chemoimmunotherapy. **(C)** H&E-stained section of the pretreatment endoscopic biopsy from the gastric mass showing adenocarcinoma with irregular infiltrative glands and nests within an inflamed stroma. **(D)** H&E-stained representative section of the treated pyloric tumor bed from the distal gastrectomy specimen showing surface ulceration abundant mixed inflammation and no viable carcinoma identified. **(E)** H&E-stained mucosal biopsy from the colon showing preserved crypt architecture, increased intraepithelial lymphocytes involving the surface and crypt epithelium, and expansion of the lamina propria by chronic inflammatory cells, compatible with lymphocytic colitis.

A previously healthy 64-year-old female presented with nausea and weight loss (**Table 2**). A CT of the abdomen and pelvis showed a cavitating mass from the gastric pylorus connecting with the pancreatic head and duodenum. A subsequent PET scan showed the distal mass was highly avid, as well as several avid pyloric and periportal lymph nodes (**Figure 2A**). Biopsy revealed gastric adenocarcinoma, pMMR, HER2-negative, CPS ≥10, and EBV-negative (**Figure 2C**).

**Table 2:** Timeline of events in case 2.

Date	Event
August 2024	CT scan showing gastric mass and EGD confirming diagnosis
September 2024	Tumor board discussion
October 2024	PET scan and started FOLFOX nivolumab
February 2025	Gastrectomy with pCR
March 2025	Experienced diarrhea with colonoscopy confirming lymphocytic colitis

Upfront surgery would have been extensive and required a total gastrectomy with likely pancreaticoduodenectomy. The case was discussed at multidisciplinary tumor boards and it was decided that upfront systemic therapy would be the most appropriate treatment. As she was determined to have unresectable disease, diagnostic laparoscopy was not completed. The patient received 3 cycles of FOLFOX and nivolumab in the first-line setting.

A follow-up PET scan showed a limited uptake and no adenopathy – it was unclear whether the findings were partial response or inflammatory changes (**Figure 2B**). Following multidisciplinary review, the patient was recommended to proceed to surgery. Staging laparoscopy was not performed before definitive resection. She subsequently received a distal gastrectomy with Roux-en-Y procedure. The fibrous tumor bed and distal stomach was entirely submitted along with 27 lymph nodes, all of which were negative for malignancy and were in keeping with granulomatous lymphadenitis (**Figure 2D**). The patient experienced diarrhea 3 months after the discontinuation of her treatment and post-resection, at which time she had a colonoscopy completed showing lymphocytic colitis (**Figure 2E**). The severity of her diarrhea was in keeping with grade 3 colitis and resolved after treatment with steroids. She was treated with prednisone 20mg with a taper of 5mg each week.

## 3 Discussion

Here we describe two patients with initially unresectable gastric cancer who achieved a pCR following treatment with upfront chemoimmunotherapy. Although neoadjuvant chemoimmunotherapy has demonstrated encouraging pCR rates in resectable gastric and GEJ cancers, reports of pCR in initially unresectable disease remain limited (15, 19). Published cases have largely involved dMMR tumors (23–25) or treatment regimens that are not approved or routinely used in our jurisdiction (26–28) (**Table 3**).

**Table 3:** Published case reports of unresectable gastric cancer who received chemoimmunotherapy and subsequently achieved a pCR.

	Gonzalez Diez et al., 2024 (23)	Sun et al., 2025 (24)	Jiang et al., 2023 (25)	Mechahougui et al., 2024 (29)	Li et al., 2021 (26)	Zhou et al., 2023 (27)	Zhu et al., 2023 (28)
Tumor primary	Gastric adenocarcinoma	Two synchronous gastric adenocarcinomas	Gastric adenocarcinoma	Gastric adenocarcinoma	GEJ adenocarcinoma	Hepatoid gastric adenocarcinoma	Gastric adenocarcinoma
Initial stage	cT4 N0 M0 (stage III)	cT4bN2M0 (stage IVA)	cT4bN2M0 (stage III)	cT4N1M1 (stage IV)	cT4N2M0 (stage III), Siewert type II	cT4aN3aMx (stage III)	pT4NxM1 (stage IV)
Resectability	Unresectable	Unresectable	Unresectable	Unresectable	Resectable, systemic therapy pursued due to patient wishes	Unclear, systemic therapy pursued due to patient wishes and MDT review	Unresectable
Molecular profile							
MMR	dMMR	dMMR	dMMR	pMMR	pMMR	pMMR	pMMR
PD-L1	NR	NR	(+)	NR	(–)	NR	(+)
CPS	100	NR	80-90	5	NR	<1	30
TPS	NR	NR	5%	NR	NR	NR	NR
TMB	NR	NR	2.25 mut/mb (low)	NR	NR	NR	20.94 mut/mb (high)
EBV	NR	NR	NR	NR	(–)	NR	(+)
Ki67	NR	NR	NR	NR	60%	80%	30%
HER2	(–)	(–)	NR	(–)	(–)	(–)	(–)
Other		Claudin 18.2 (–)	Overrepresentation of gut microbiota strains such as *Akkermansia muciniphila*, *Bifidobacterium longum*, *Odoribacter*, and *Veillonella*				PIK3CB
Neoadjuvant systemic therapy	Initial FLOT Then FOLFOX- nivolumab	SOX + tislelizumab	S1 + apatinib + abraxane + camrelizumab	FOLFOX + nivolumab	SOX + camrelizumab	SOX + terelizumab	XELOX + tislelizumab
Preoperative duration of systemic therapy	6 cycles	3 cycles	4 cycles of S1 + apatinib + abraxane + camrelizumab, then 2 cycles S-1	4 cycles	3 cycles	3 cycles	8 cycles
Surgery	Subtotal gastrectomy + D2 lymphadenectomy + omentectomy + segmental resection of the splenic flexure	Distal gastrectomy + D2 lymphadenectomy	Radical gastrectomy + the lower esophagus, part of the left diaphragm D1 lymphadenectomy	Radical gastrectomy + D2 lymphadenectomy + liver metastectomy	Radical gastrectomy + D2 lymphadenectomy	Radical gastrectomy + D2 lymphadenectomy	Radical gastrectomy and esophago-jejunostomy
Postoperative pathology	Complete pathologic response	Pathologic complete response	Pathologic complete response	Pathologic complete response	Pathologic complete response	Pathologic complete response	Pathologic complete response
Postoperative systemic therapy	Capecitabine + nivolumab x 12 months	Not reported	None	Nivolumab x 6 months	3 cycles of SOX + camrelizumab	None	Planned: 4–5 cycles of XELOX + Tislelizumab followed by tislelizumab monotherapy
Follow up	12 months	Not reported	19 months	12 months	5 months	12 months	4 months

CPS, combined positive score; EBV, Epstein bar virus; FOLFOX, 5FU, leucovorin, oxaliplatin; HER2, human epidermal growth factor receptor 2; Mut/mb, mutations/megabase; NR, not reported; SOX, S1, oxaliplatin; TMB, tumor mutational burden; TPS, tumor proportion score; XELOX, capecitabine, oxaliplatin.

To our knowledge, only one prior case report has described a pMMR unresectable gastric cancer patient who achieved pCR following chemoimmunotherapy (29). The patient described in Mechahougui et al. (29) had unresectable T4N1M1 gastric adenocarcinoma that was pMMR, HER2-negative, and with a CPS of 5 (indicative of moderate PD-L1 expression). After receiving four cycles of FOLFOX plus nivolumab, the patient underwent radical gastrectomy, D2 lymphadenectomy, and liver metastasectomy, with final pathology demonstrating pCR. Postoperatively, the patient received six months of nivolumab and remained disease-free after 12 months of follow-up at the time of publication. In contrast, both of our patients received three doses of nivolumab prior to surgery, had high PD-L1 expression (CPS ≥10), and neither received postoperative systemic therapy.

Although several perioperative trials have demonstrated impressive pCR rates with chemoimmunotherapy, evidence of a survival benefit has only recently begun to emerge and its applicability to initially unresectable disease remains uncertain. KEYNOTE-585 (17) reported an improved pCR rate (12.9%) but did not initially show a significant survival benefit. A recent interim subgroup analysis found EFS and OS were not reached among those receiving FLOT as the chemotherapy backbone, which is promising (18). More recently, MATTERHORN reported a pCR rate of 19.2% and found an EFS improvement and potentially an OS improvement after 12 months for those receiving perioperative FLOT with durvalumab (20). In comparison, perioperative chemotherapy alone has achieved pCR rates of 11.7-16% in the SPACE-FLOT and FLOT4 studies, respectively (2, 30). Importantly, these trials enrolled patients with resectable disease, whereas both of our patients were considered unresectable at diagnosis.

Our patients received FOLFOX and nivolumab. FOLFOX was selected because FLOT-based chemotherapy is not a standard accessible treatment for unresectable gastric cancer in Canada. Consequently, the applicability of perioperative FLOT-based trials to our patients is uncertain. Studies comparing FLOT and FOLFOX head-to-head are limited. The recently published PRODIGE 51-FFCD-GASTFOX compared a modified FLOT regimen (TFOX), with FOLFOX among patients with advanced HER2-negative gastric or GEJ adenocarcinoma in the first line. They demonstrated an improved median OS (15.08 months in the TFOX versus 12.65 months in the FOLFOX group; HR 0·82 [0·68-0·99]; P = 0·048 (31);). However, treatment-related adverse events were much higher in the TFOX group (27% vs 13%), making this regimen inappropriate for our patients.

Both of our patients had tumors with high PD-L1 CPS (≥10). Trials of first-line chemoimmunotherapy for gastric and GEJ cancer in the metastatic setting (9–11, 32) have shown survival benefits primarily in enriched populations (e.g. CPS ≥ 1, or PD-L1 ≥ 1%). It is unclear whether our patients would have had such a profound response if they did not have CPS ≥10 tumors.

Our first patient had EBV-associated gastric cancer (EBVaGC), a distinct subtype accounting for 10% of gastric cancer, with favorable prognosis (33), dense immune infiltration, extensive DNA hypermethylation, and PD-L1 overexpression (33, 34). These features provide a biologic rationale for the benefit of ICIs in these patients, although clinical evidence of this remains limited. Bai et al. found comparable PFS and OS amongst EBV positive pMMR and dMMR patients when treated with immunotherapy (22), but a trend towards improved survival with combination ICI therapy was nonsignificant. Other studies have suggested higher response rates in PD-L1-positive EBVaGC, albeit with substantial variability (33–35). Indeed, our EBV-positive patient also had a PD-L1 CPS of ≥10 (35). However, our second patient was EBV-negative and also achieved a pCR, suggesting that EBV was not the primary driver of ICI response among our patients.

EBV positivity may be relevant in patients with intermediate PD-L1 expression (CPS1-9), where the benefit of immunotherapy appears to be less certain (22, 34). Further, although EBVaGC is typically also enriched for PIK3CA and ARID1A mutations (9) (33, 36), the clinical utility of these remain investigational (37, 38), and testing for this was not performed in our patients.

Both of our patients experienced an immunotherapy-related adverse event (ir-AE) – hepatitis and lymphocytic colitis, respectively. Whether ir-AEs indicate treatment efficacy is an area of ongoing research. In a systematic review and meta-analysis evaluating the relationship between ir-AEs and clinical outcomes among solid tumor malignancies (39), progression-free survival was significantly longer for gastric and GEJ cancer patients who had an ir-AE, although the absolute number of patients was small. All patients analyzed were PD-L1 ≥1. It is possible that the ir-AEs experienced by our patients were indicative of a favourable treatment response, which may be due to a more robust immune response among these patients. Notably, neither of these ir-AEs were an impediment to surgery.

This report has several limitations. Neither patient underwent next-generation sequencing or additional tumor profiling (e.g. tumor mutational burden, CD8 testing, PIK3CA or ARID1A mutations), as these investigations are not standard of care in our jurisdiction and were unlikely to yield clinically actionable findings. In addition, our second patient did not undergo staging laparoscopy. This was initially deferred because imaging demonstrated unresectable disease, and the findings were unlikely to alter management. However, once the patient became a surgical candidate, staging laparoscopy should have been reconsidered to assess for radiographically occult peritoneal metastases.

These cases highlight the potential for reassessing resectability during systemic therapy. They underscore the importance of ongoing multidisciplinary evaluation and may inform discussions held with patients regarding the possibility of conversion surgery in select cases. In both cases, surveillance was chosen after surgery because of prior ir-AEs. Adjuvant FLOT was considered but not administered, given the absence of evidence to support its use following neoadjuvant FOLFOX plus nivolumab. More broadly, there is limited evidence to guide the use of adjuvant chemoimmunotherapy or optimal surveillance strategies after resection in this setting.

These cases illustrate that upfront chemoimmunotherapy can, in some instances, achieve a pCR in initially unresectable disease. Whether a subset of patients with an exceptional response could ultimately avoid surgery remains unknown. Improved methods to identify pCR without resection, such as imaging, endoscopy, or circulating tumor DNA, warrant further investigation and may help reduce treatment-related morbidity in carefully selected patients.

## 4 Conclusions

We describe two patients with PD-L1-positive, locally advanced unresectable gastric cancer who were initially treated with palliative intent chemo-immunotherapy after being considered unsuitable for curative treatment. Both subsequently became candidates for curative resection and achieved a pCR. Although limited by the nature of a two-patient case report, these cases suggest that chemoimmunotherapy may, in rare instances, facilitate conversion to resectability in initially unresectable disease. They also highlight important unanswered questions regarding the role of chemoimmunotherapy in the perioperative setting, its impact on long-term outcomes, and the optimal postoperative management of patients who achieve a pCR following curative resection.

## 5 Patient perspective

When I was first diagnosed with stomach cancer, the doctors told me I had 6 months to live. They told me I should go home and “put my ducks in a row”. Indeed, I planned my own funeral. Before I started chemoimmunotherapy, I was scared and anxious and felt a bit overwhelmed. Starting treatment was a huge learning curve, I had no idea how my body would react. Once I started treatment, the ball started rolling fast. You can’t compare the feeling of treatment to anything, it was a unique new experience and I felt so crummy. But then my CT scan showed that the cancer was getting smaller and smaller. I thought “what a blessing, I’m being given a miracle”. It made me optimistic there was some hope. It gave me a fighting chance. I never thought I would go from being inoperable to operable, and for that I thank the Lord, my supportive friends, my medical team, and the treatment. I wouldn’t wish this experience on anyone but I’m very pleased with the experience I’ve had.

## Data Availability

The original contributions presented in the study are included in the article/**Supplementary Material**. Further inquiries can be directed to the corresponding authors.
